# Author Correction: High-latitude biomes and rock weathering mediate climate–carbon cycle feedbacks on eccentricity timescales

**DOI:** 10.1038/s41467-021-21827-8

**Published:** 2021-03-03

**Authors:** David De Vleeschouwer, Anna Joy Drury, Maximilian Vahlenkamp, Fiona Rochholz, Diederik Liebrand, Heiko Pälike

**Affiliations:** 1 MARUM - Center for Marine and Environmental Sciences, University of Bremen, Klagenfurterstraße 2-4, 28359 Bremen, Germany; 2 Department of Earth Sciences, University College London, Gower Street, London, WC1E 6BT UK; 3 Present Address: Research Group for Earth Observation, Pädagogische Hochschule Heidelberg, Czernyring 22/10-12, 69120 Heidelberg, Germany

**Keywords:** Palaeoceanography, Palaeoclimate

Correction to: *Nature Communications* 10.1038/s41467-020-18733-w, published online 6 October 2020.

The original version of this Article contained an error in Fig. 1, in which the Pliocene–Pleistocene boundary was incorrectly placed at 1.8 Ma. The correct date of the Pliocene–Pleistocene boundary is 2.59 Ma. This has now been corrected in the PDF and HTML versions of the Article.

