# Supplementary material for: High-latitude biomes and rock weathering mediate climate–carbon cycle feedbacks on eccentricity timescales
Source: Nat Commun. 2020 Oct 6;11:5013. doi: 10.1038/s41467-020-18733-w (PMC7538577; doi:10.1038/s41467-020-18733-w)
Supplement: Supplementary file 1 — Supplementary Information [file 41467_2020_18733_MOESM1_ESM.pdf]

Supplementary Information

High-latitude biomes and rock weathering mediate climate – carbon cycle feedbacks on eccentricity timescales

David De Vleeschouwer[1*], Anna Joy Drury[1,2], Maximilian Vahlenkamp[1], Fiona Rochholz[1,3], Diederik Liebrand[1], Heiko Pälike[1]

[1] MARUM - Center for Marine and Environmental Sciences, University of Bremen, Klagenfurterstraße 2-4, Bremen, 28359, Germany.
[2] Currently at: Department of Earth Sciences, University College London, Gower Street, London, WC1E 6BT, UK.
[3] Currently at: Research Group for Earth Observation, Pädagogische Hochschule Heidelberg, Czernyring 22/10-12, Heidelberg, 69120, Germany

*e-mail: ddevleeschouwer@marum.de

Site	Lat.	Lon.	Source	Ocean	Source of Age-Depth Model	Start (Ma)	Stop (Ma)
**926**	3.7°S	42.9°W	http:// doi.org/10.1594/PANGAEA.743431	North Atlantic	Pälike et al.[1]	**20.215**	**22.56**
**926**	3.7°S	42.9°W	https://doi.org/10.1594/PANGAEA.870873	North Atlantic	Wilkens et al.[2] & Zeeden et al.[3]		
**982**	57.5°N	15.9°W	https://doi.org/10.1594/PANGAEA.884293	North Atlantic	Drury et al.[4]	**5.174**	**6.677**
**1090**	42.9°S	8.9°E	http:// doi.org/10.1594/PANGAEA.708128	South Atlantic	Billups et al.[5]	**20.025**	**20.215**
**1090**	42.9°S	8.9°E	http:// doi.org/10.1594/PANGAEA.708128	South Atlantic	Billups et al.[5]	**22.56**	**23.9**
**1146**	19.5°N	116.3°E	http:// doi.org/10.1594/PANGAEA.825480	Pacific	Revision in Ext. Data Table 2	**7.891**	**12.834**
**1218**	8.9°N	135.4°E	http:// doi.org/10.1594/PANGAEA.547797	Pacific	Pälike et al.[6]	**23.9**	**35.0**
**1264**	28.6°S	2.6°E	http://www1.ncdc.noaa.gov/pub/data/paleo/contributions_by_author/bell2014/bell2014-1264.txt	South Atlantic	Bell et al.[7]	**3.2844**	**5.174**
**1264 / 1265**	28.6°S	2.6°E	https://doi.org/10.1594/PANGAEA.862585	South Atlantic	Liebrand et al.[8]		
**1267**	28.1°S	1.7°E	http://www1.ncdc.noaa.gov/pub/data/paleo/contributions_by_author/bell2014/bell2014-1267.txt	South Atlantic	Bell et al.[7]	**0**	**3.284**
**U1334**	8.0°N	132.0°W	https://doi.org/10.1594/PANGAEA.885365	Pacific	Beddow et al.[9]		
**U1337**	3.8°N	123.2°W	https://doi.org/10.1594/PANGAEA.872508	Pacific	Drury et al.[10]	**6.677**	**7.891**
**U1337**	3.8°N	123.2°W	http:// doi.org/10.1594/PANGAEA.839743	Pacific	Holbourn et al.[11]	**15.800**	**20.025**
**U1337**	3.8°N	123.2°W	https://doi.org/10.1016/j.epsl.2018.07.025	Pacific	Tian et al.[12]		
**U1338**	2.5°N	118.0°W	http:// doi.org/10.1594/PANGAEA.820095	Pacific	Holbourn et al.[13]	**12.834**	**15.800**
**U1338**	2.5°N	118.0°W	https://doi.pangaea.de/10.1594/PANGAEA.856679	Pacific	Drury et al.[14]		

Supplementary Table 1: Overview of all individual sedimentary archives with their astronomical age models that are used in this study to either construct the megasplice or to calculate phase differences on eccentricity time-scales. The Start (Ma) and Stop (Ma) indicate the ages at which the megasplice jumps from one record to the next.

Site	Hole	Core	Type	Section	Interval	MBSF	MCD	RMCD	Age	Comment
1146	C	30	X	2	10	283.4	299.7	299.7	5.12	Holbourn et al., 2018
1146	C	30	X	3	18	284.98	301.28	301.28	5.178	Holbourn et al., 2018
1146	C	30	X	4	88	287.18	303.48	303.48	5.259	Holbourn et al., 2018
1146	A	32	X	3	53	293.33	311.78	311.78	5.549	Holbourn et al., 2018
1146	A	32	X	4	103	295.33	313.78	313.78	5.59	Retuned (using 926 and U1337 data)
1146	A	32	X	5	128	297.08	315.53	315.53	5.642	Retuned (using 926 and U1337 data)
1146	A	32	X	6	73	298.03	316.48	316.48	5.696	Additional tie (using 926 data)
1146	A	32	X	0	38	299.47	317.92	317.92	5.751	Holbourn et al., 2018
1146	A	33	X	1	103	300.53	319.28	319.28	5.793	Holbourn et al., 2018
1146	A	33	X	5	48	305.98	324.73	324.73	5.992	Retuned (using 926 and U1337 data)
1146	A	33	X	6	138	308.38	327.13	327.13	6.059	Retuned (using 926 and U1337 data)
1146	A	34	X	4	14	313.74	332.69	332.69	6.259	Retuned (using U1337 data)
1146	C	34	X	4	8	319.88	337.73	337.73	6.37	Retuned (using U1337 data)
1146	C	34	X	4	117	320.97	338.82	338.82	6.424	Retuned (using U1337 data)
1146	A	35	X	3	80	322.5	342.2	342.2	6.554	Retuned (using U1337 data)
1146	A	35	X	5	81	325.51	345.21	345.21	6.674	Retuned (using U1337 data)
1146	C	35	X	5	94	331.84	350.74	350.74	6.875	Retuned (using U1337 data)
1146	A	36	X	5	78	335.18	357.28	357.28	6.985	Retuned (using U1337 data)
1146	C	36	X	5	3	340.63	362.28	362.28	7.155	Retuned (using U1337 data)
1146	A	37	X	5	113	345.23	367.98	367.98	7.322	Retuned (using U1337 data)
1146	C	37	X	4	38	349.08	372.18	372.18	7.438	Retuned (using U1337 data)
1146	A	38	X	3	123	351.93	375.68	375.68	7.587	Retuned (using U1337 data)
1146	C	38	X	1	48	354.28	378.43	378.43	7.682	Retuned (using U1337 data)
1146	A	39	X	2	38	359.18	382.98	383.78	7.851	Retuned (using U1337 data)
1146	A	39	X	4	67	362.47	386.27	387.07	7.999	Retuned (using U1337 data)
1146	A	39	X	6	16	364.96	388.76	389.56	8.065	Retuned (using U1337 data)
1146	C	39	X	4	6	368.06	393.36	394.16	8.203	Retuned (using U1337 data)
1146	A	40	X	4	143	372.83	397.63	398.43	8.404	Retuned (using ET curve)
1146	C	40	X	1	68	373.78	399.44	400.24	8.461	Retuned (using ET curve)
1146	C	40	X	2	118	375.78	401.44	402.24	8.544	Holbourn et al., 2018
1146	A	41	X	2	78	378.78	404.62	405.42	8.664	Holbourn et al., 2018
1146	A	41	X	5	103	383.53	409.37	410.17	8.868	Holbourn et al., 2018
1146	C	41	X	1	121.25	384.01	410.87	411.67	8.953	Holbourn et al., 2018
1146	C	41	X	3	36.25	386.16	413.02	413.82	9.034	Holbourn et al., 2018
1146	A	42	X	2	103	388.63	414.87	415.67	9.121	Holbourn et al., 2018
1146	A	42	X	3	48	389.58	415.82	416.62	9.157	Holbourn et al., 2018
1146	A	42	X	4	58	391.18	417.42	418.22	9.24	Holbourn et al., 2018
1146	C	42	X	2	3	395.43	420.92	421.72	9.403	Holbourn et al., 2018
1146	C	42	X	5	14	398.54	424.03	424.83	9.521	Holbourn et al., 2018
1146	A	43	X	6	13	403.33	429.72	430.52	9.762	Holbourn et al., 2018
1146	A	44	X	2	3	406.83	433.22	434.02	9.882	Holbourn et al., 2018
1146	A	44	X	4	70	410.5	436.89	437.69	10.024	Holbourn et al., 2018
1146	A	44	X	5	84	412.14	438.53	439.33	10.107	Retuned (using ET curve)
1146	C	44	X	3	127	415.87	441.51	442.31	10.271	Retuned (using ET curve)
1146	C	44	X	4	130	417.4	443.04	443.84	10.349	Retuned (using ET curve)
1146	A	45	X	3	7	417.97	444.71	445.51	10.457	Retuned (using ET curve)
1146	C	45	X	3	133	425.53	451.57	452.37	10.7	Retuned (using ET curve)
1146	C	45	X	5	3	427.23	453.27	454.07	10.801	Retuned (using ET curve)
1146	A	46	X	3	102	428.52	456.76	457.56	10.942	Retuned (using ET curve)
1146	A	46	X	4	100	430	458.24	459.04	11.022	Retuned (using ET curve)
1146	C	46	X	4	139	436.49	465.13	465.93	11.31	Retuned (using ET curve)
1146	A	47	X	3	10	437.2	467.44	468.24	11.411	Retuned (using ET curve)
1146	A	47	X	5	8	440.18	470.42	471.22	11.576	Retuned (using ET curve)
1146	A	47	X	5	150	441.6	471.84	472.64	11.656	Retuned (using ET curve)
1146	C	47	X	3	22	443.42	473.11	473.91	11.719	Retuned (using ET curve)
1146	C	47	X	4	81	445.51	475.2	476	11.797	Holbourn et al., 2018
1146	A	48	X	2	134	446.54	477.07	477.87	11.898	Holbourn et al., 2018
1146	A	48	X	4	48	448.68	479.21	480.01	11.998	Holbourn et al., 2018
1146	A	48	X	6	46	451.66	482.19	482.99	12.14	Holbourn et al., 2018
1146	C	48	X	3	66	453.46	483.37	484.17	12.22	Retuned (using ET curve)
1146	C	48	X	4	35	454.65	484.56	485.36	12.298	Retuned (using ET curve)
1146	C	48	X	5	113	456.93	486.84	487.64	12.38	Retuned (using ET curve)
1146	A	49	X	4	138	459.33	488.79	489.59	12.48	Retuned (using ET curve)
1146	C	49	X	3	139	463.89	493.2	494	12.686	Retuned (using U1338 data)
1146	C	49	X	6	46	467.46	496.77	497.57	12.86	Retuned (using U1338 data)
1146	C	49	X	CC	15	469.12	498.43	499.23	12.96	Retuned (using U1338 data)

Supplementary Table 2: Revision of Site 1146 age model, based on the revised meter composite scale (rmcd) presented in Holbourn et al. 2018[15]. mbsf = meter below sea floor. mcd = meter composite depth. Tuning targets are Site 926 and Site U1337 $\delta^{13}C_{benthic}$ data from Drury et al.[10], Site U1338 $\delta^{13}C_{benthic}$ data from Holbourn et al.[13], and an eccentricity-tilt (ET) composite constructed based on astronomical solution La2004[16].

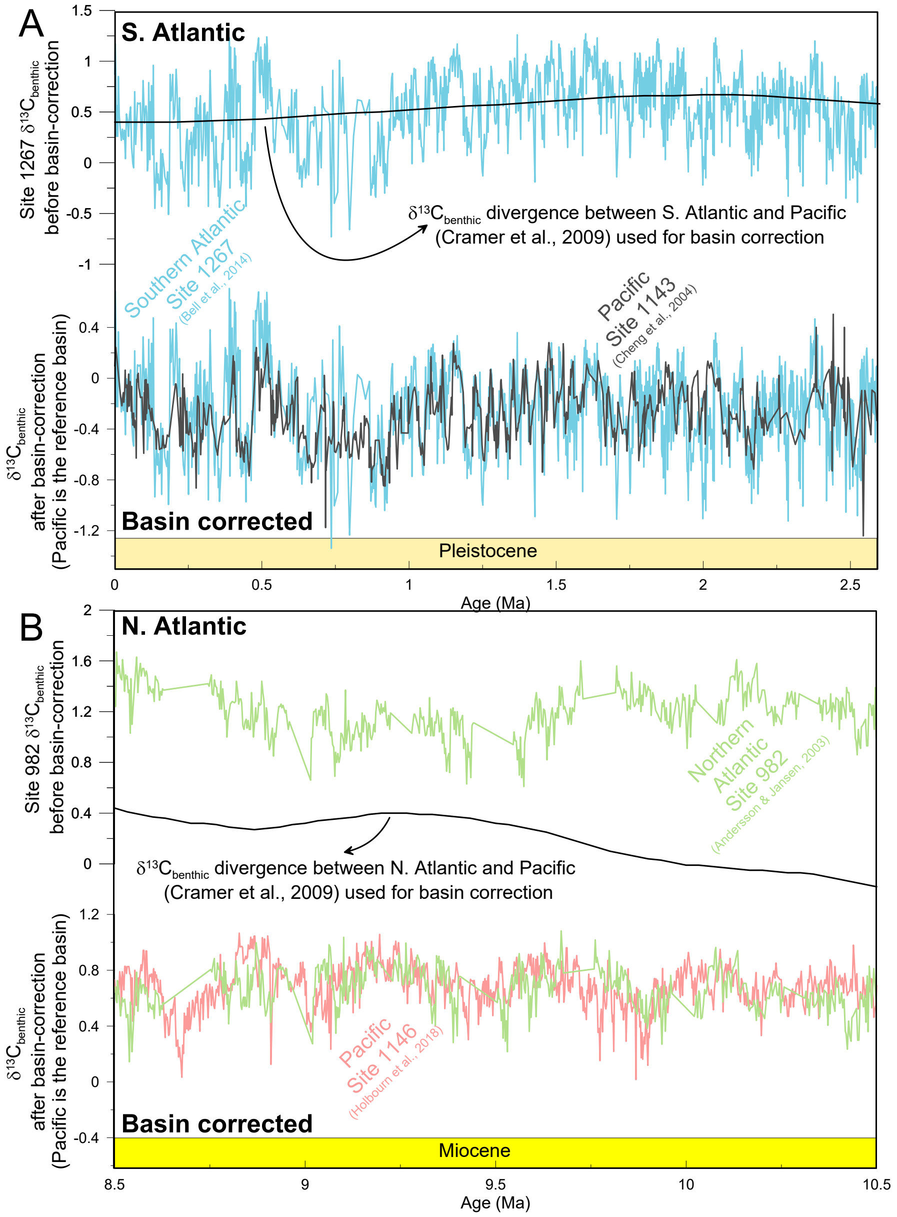

Supplementary Figure 1: An inter-ocean comparison of $\delta^{13}C_{benthic}$ records and their astronomical imprint. The uncorrected Atlantic Sites 1267 and 982 $\delta^{13}C_{benthic}$ records[7,17] are shown, together with the deep-ocean $\delta^{13}C$ divergence trend between the North or South Atlantic and the Pacific, as estimated by Cramer et al.[18]. We use this trend for calculating the basin-corrected $\delta^{13}C_{benthic}$ records, with the Pacific Ocean as the reference basin. The basin-corrected $\delta^{13}C_{benthic}$ record are then compared to contemporaneous Pacific (South China Sea) $\delta^{13}C_{benthic}$ records[15,19]. All records exhibit a similar response to astronomical forcing despite their geographic separation, we refer to this characteristic as a global $\delta^{13}C_{benthic}$ heartbeat. Sites 1267 and 1146 $\delta^{13}C_{benthic}$ records are used in the megasplice as they are of higher temporal resolution.

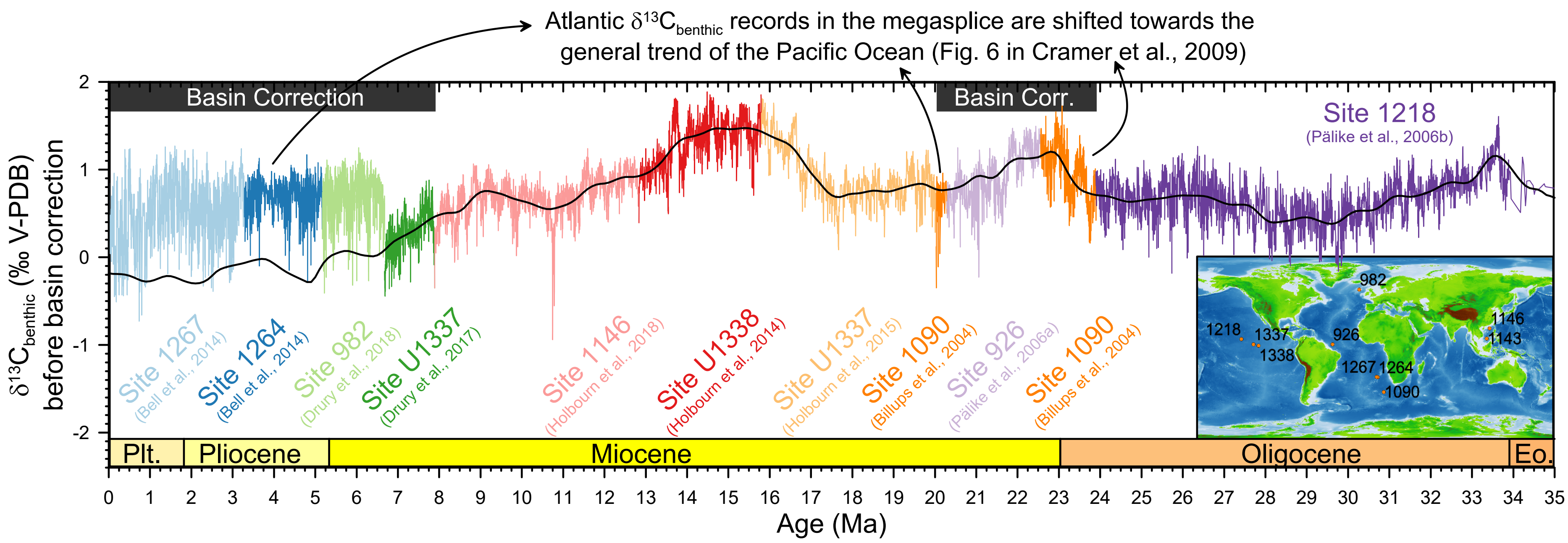

Supplementary Figure 2: Benthic δ^{13}C megasplice before basin correction. During basin correction, Atlantic δ$^{13}C_{benthic}$ records are shifted vertically (along the δ^{13}C y-axis) to match the general trend of the Pacific Ocean (Figure 6 in Cramer et al.[18]) and to obtain the benthic δ^{13}C megasplice as shown in Figure 1. The general δ^{13}C trend of the Pacific Ocean only consists of very low-frequency variability and thus does not introduce any features that might interfere with our analyses in the *Milankovitch* frequency band (20 – 405 kyr). World map after Amante and Eakins[20].

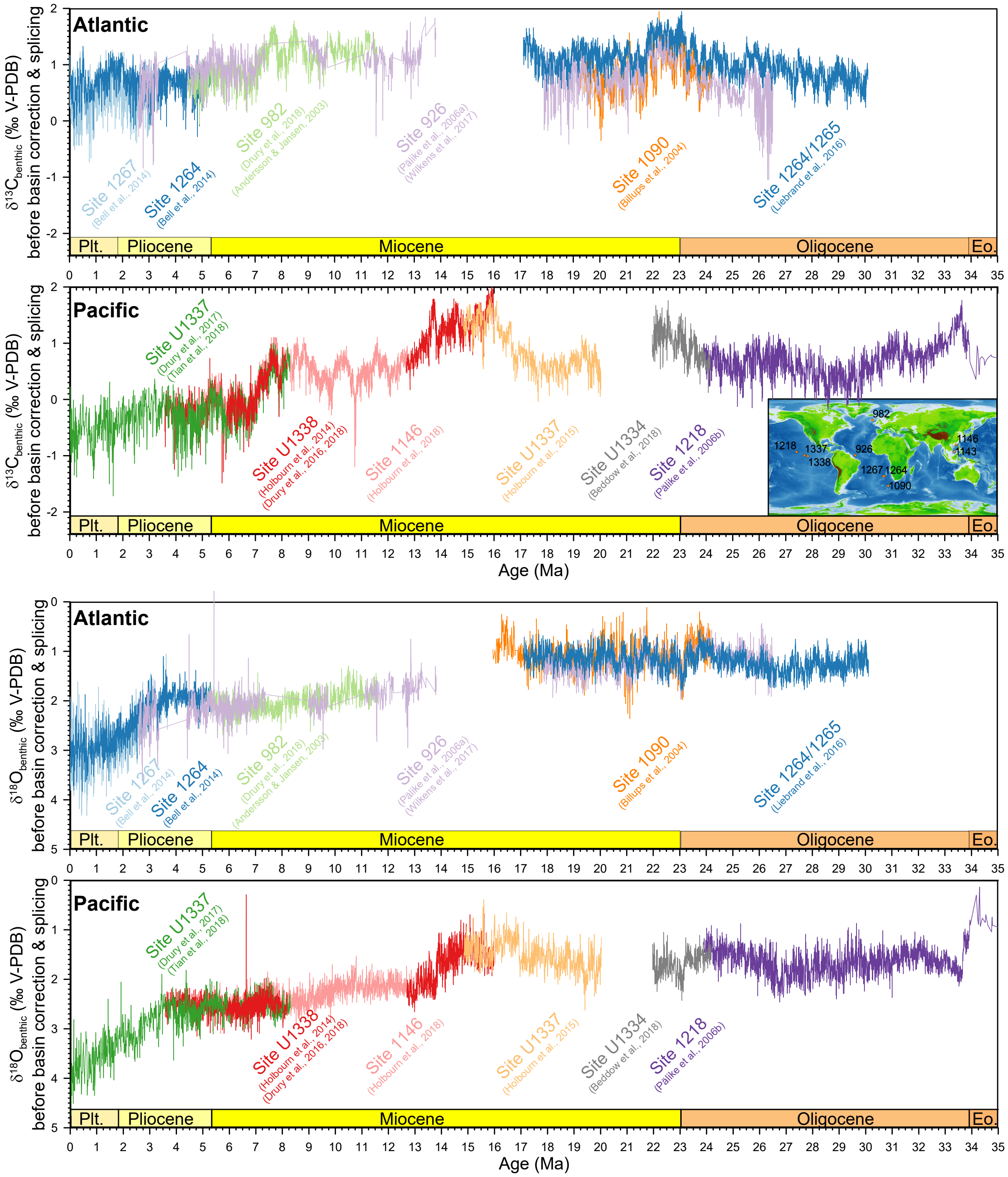

Supplementary Figure 3: Overview of all δ^{13}C and δ^{18}O datasets that are subjected to phase analysis (results in Figures 2). All panels show the raw isotopic data before any correction (equilibrium correction or basin correction) or splicing between sites. The splicing step is illustrated in Supplementary Figure 4. World map after Amante and Eakins[20].

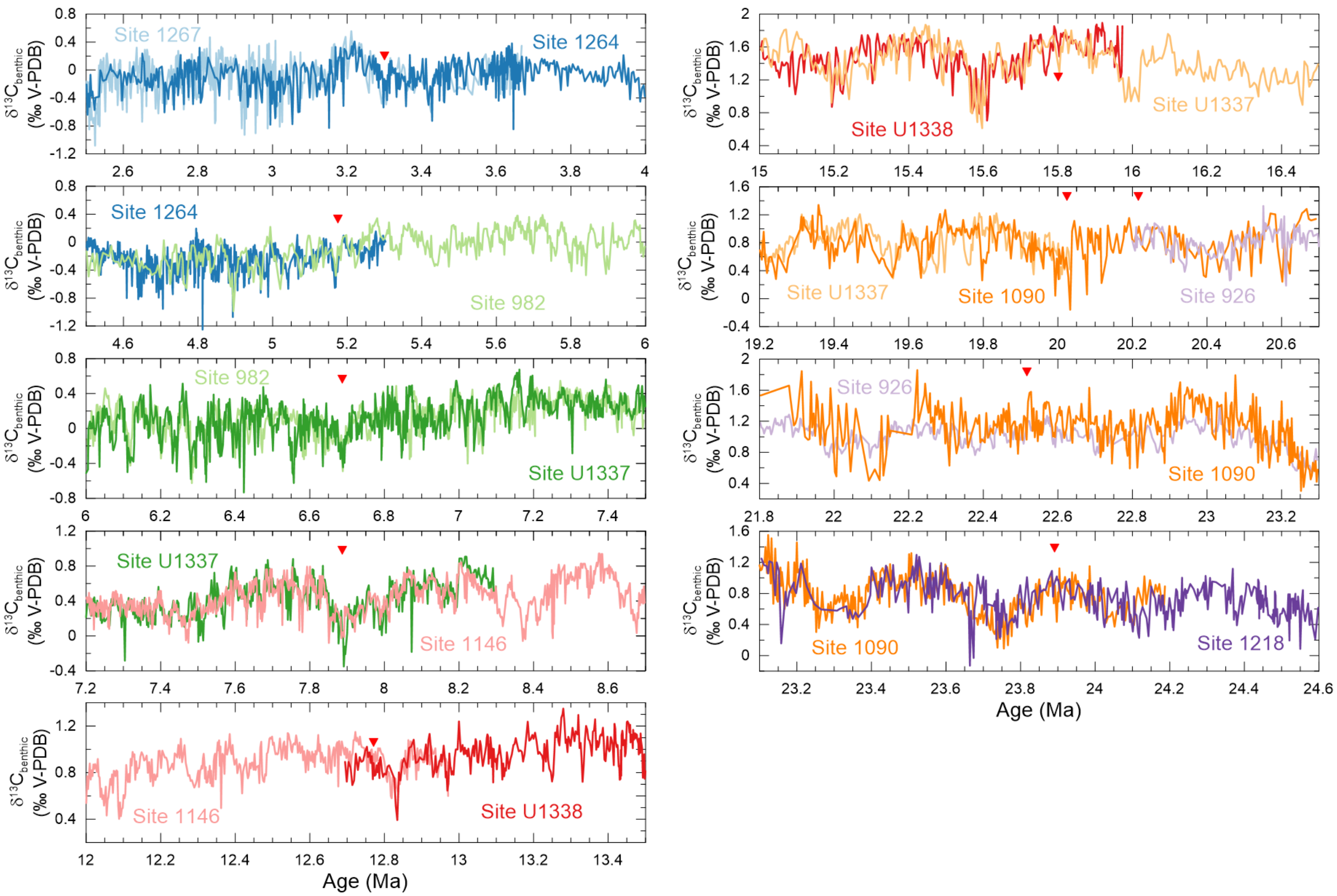

Supplementary Figure 4: Correlation between the different records used in the megasplice (after basin-correction). Red triangles indicate the stratigraphic positions at which the megasplice passes from one record to another.

Frequency range for phase analysis	Interval I	Interval II
100 – 125 kyr	R = 0.338<br>*p*-value = 0.176	R = -0.277<br>*p*-value = 0.032
95 – 130 kyr	R = 0.360<br>*p*-value = 0.15	R = -0.215<br>*p*-value = 0.037
**90 – 135 kyr**	**R = 0.399**<br>***p*-value = 0.060**	**R = -0.240**<br>***p*-value = 0.015**
85 – 140 kyr	R = 0.323<br>*p*-value = 0.134	R = -0.160<br>*p*-value = 0.074
80 – 145 kyr	R = 0.319<br>*p*-value = 0.113	R = -0.166<br>*p*-value = 0.059
75 – 150 kyr	R =0.232<br>*p*-value = 0.266	R = - 0.073<br>*p*-value = 0.408

Supplementary Table 3: Sensitivity of correlation analysis in Interval I and II to different frequency ranges for phase analysis. Too narrow frequency ranges make that some eccentricity-related variability is not considered for phase analysis, negatively impacting results. Too wide frequency ranges allow for non-eccentricity-related variability to be considered during phase analysis, also negatively impacting results. The 90 – 135 kyr frequency window is used in the main manuscript to calculate phase relationships between $\delta^{13}C$ and $\delta^{18}O$ on time-scales of ~100-kyr eccentricity (see also Supplementary Figure 5). We choose this frequency window to include the four main ~100-kyr terms[21] [(g_4-g_5) with 94.9 kyr period, (g_3-g_5) with 98.9 kyr period, (g_4-g_2) with 123.9 kyr period, (g_3-g_2) with 130.7 kyr period] and accommodates minor (<5%) age-model inaccuracies.

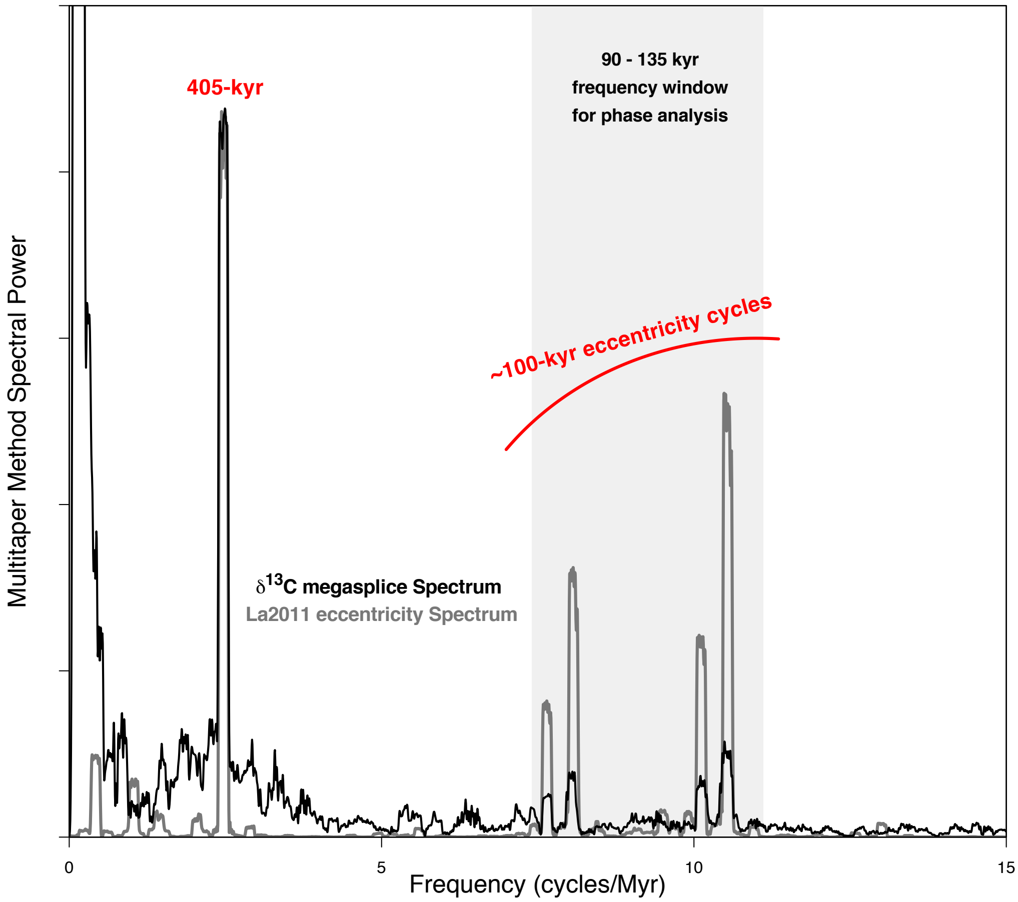

Supplementary Figure 5: Motivation for choosing a 90 – 135 kyr frequency window for phase analysis. We calculate phase relationships between $\delta^{13}C$ and $\delta^{18}O$ on time-scales of ~100-kyr eccentricity within 90 – 135 kyr frequency windows. We choose this frequency range to encompass all major ~100-kyr eccentricity components in the La2011 eccentricity solution[21,22] [(g_4-g_5) with 94.9 kyr period, (g_3-g_5) with 98.9 kyr period, (g4-g2) with 123.9 kyr period, (g_3-g_2) with 130.7 kyr period], and at the same time accommodates minor (<5%) age-model inaccuracies. These four main ~100 kyr eccentricity components can also be discerned in the power spectrum of the $\delta^{13}C_{benthic}$ megasplice, indicating the fundamental coupling between eccentricity and deep-sea carbon isotopes throughout the last 35 Ma.

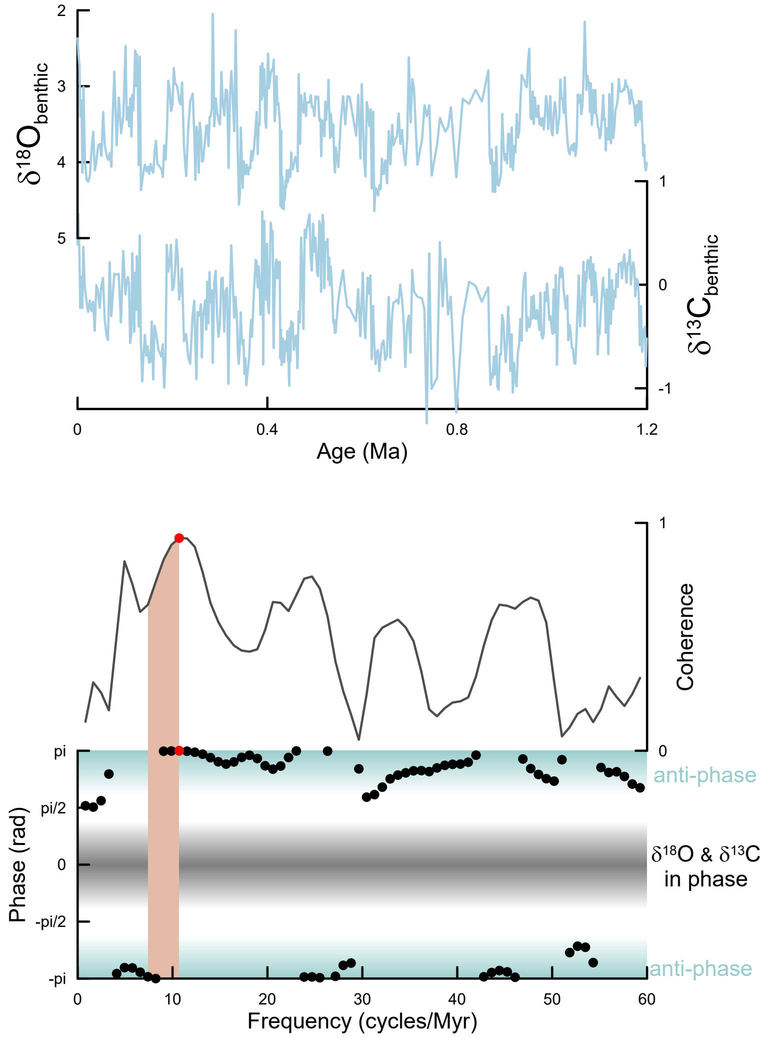

Supplementary Figure 6: Illustration of phase-analysis within 1.2-Myr wide windows. Cross-spectral analysis is carried out between $\delta^{13}C$ and $\delta^{18}O$ time-series. Subsequently, the frequency with the highest coherence within the 90 – 135 kyr frequency window is selected. The phase between $\delta^{13}C$ and $\delta^{18}O$ at that specific frequency is retained for further analysis in Figures 2 and 3.

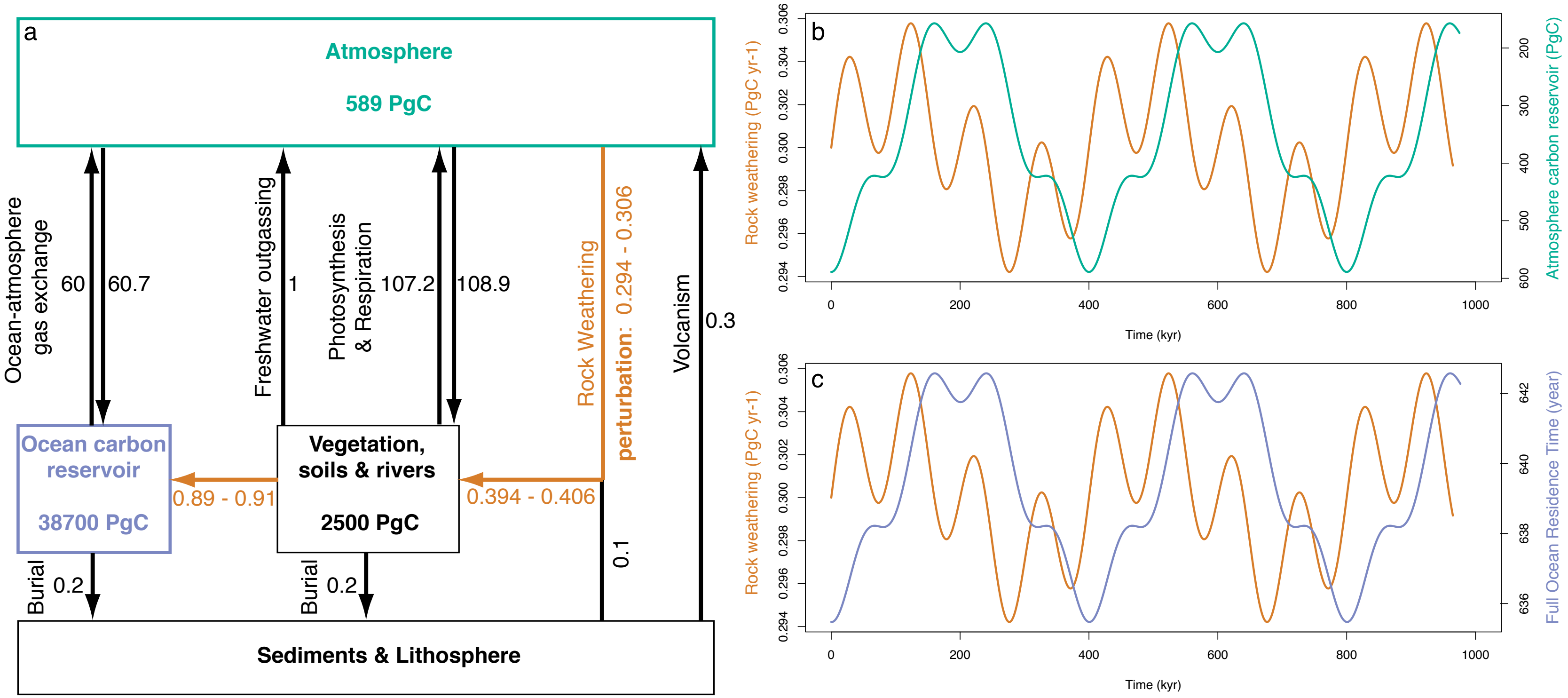

Supplementary Figure 7: The effect of a perturbed rock weathering atmospheric CO_2 sink in a simple carbon cycle box model. (a) Box model with carbon stocks and fluxes according to Figure 6.1 in Ciais et al.[23]. The rock weathering carbon flux is perturbated with a sinusoid with a periodicity of 100 and 400 kyr, representative of eccentricity forcing. The perturbation of the rock weathering flux ranges between 0.294 and 0.306 PgC yr^{-1}. The perturbed weathering C flux is propagated into the C flux through continental run-off, yet all other C fluxes in the box model were kept constant. **(b)** Rock weathering variability (orange) causes threefold fluctuations in the size of the reservoir between 190 and 600 PgC (and thus threefold fluctuation in atmospheric CO_2 concentration, green). **(c)** Rock weathering variability (orange) only induces ~1% fluctuations in the residence time of carbon in the ocean reservoir (blue).

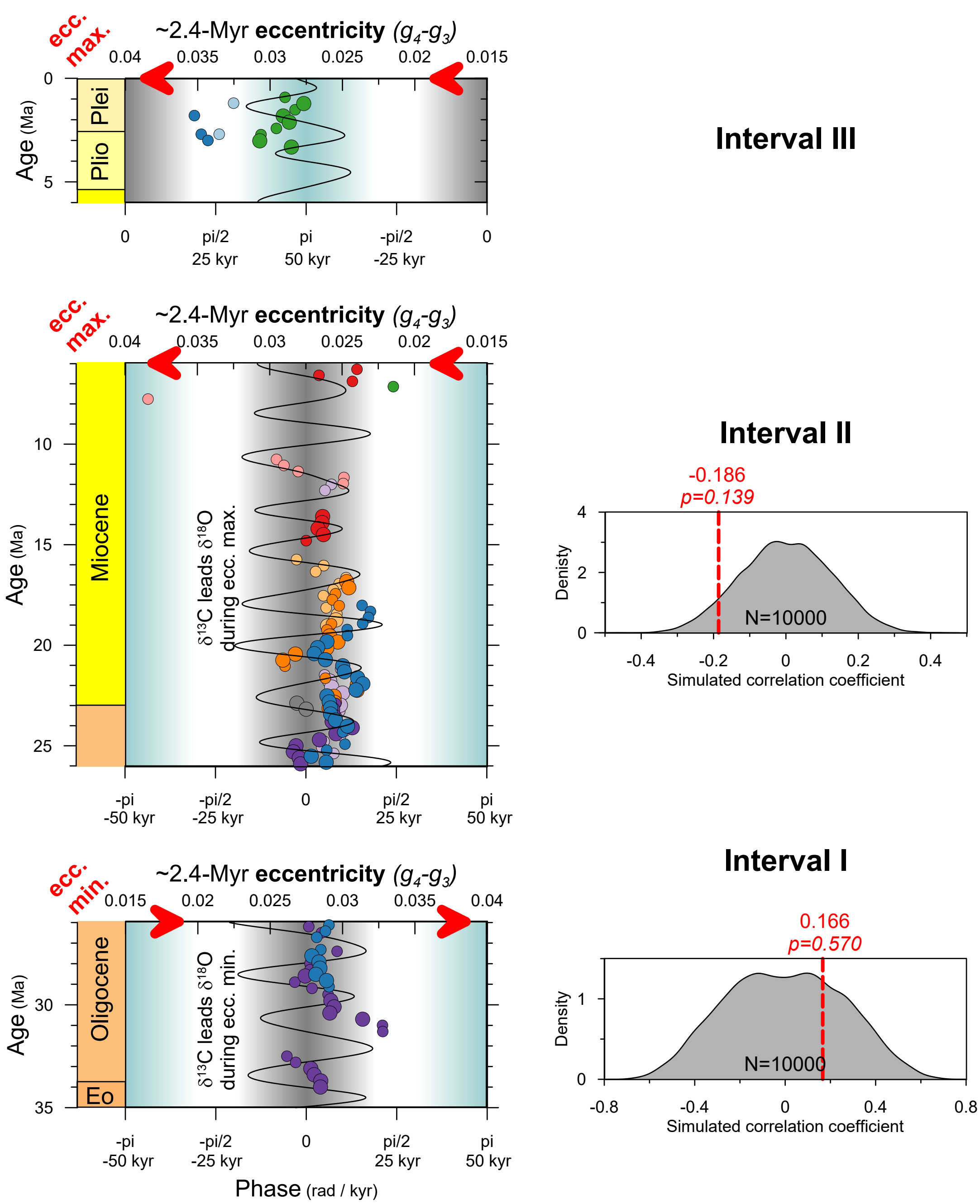

Supplementary Figure 8: Long 2.4-Myr eccentricity *(g_4-g_3)* modulates Oligocene and Miocene leads and lags between δ^{13}C and δ^{18}O on 405-kyr eccentricity time-scales. Prior to 26 Ma (Interval I), long 2.4-Myr eccentricity minima correlate with time-intervals when δ^{13}C leads δ^{18}O (positive Spearman correlation coefficient of 0.360 between phase and eccentricity, albeit not statistically significant with a *p*-value of 0.27). Between 26 and 6 Ma (Interval II), long 2.4-Myr eccentricity minima correlate with time intervals when δ^{18}O leads δ^{13}C (negative Spearman correlation coefficient of -0.240). After 6 Ma (Interval III), the correlation between 2.4-Myr eccentricity and phase ceases (R = -0.224, not statistically significant). This figure only displays phase results for analyses with coherence > 0.3 within the 370 – 435 kyr frequency window. Color coding of the different sites is identical to Figure 2.

Supplementary References

1 Pälike, H., Frazier, J. & Zachos, J. C. Extended orbitally forced palaeoclimatic records from the equatorial Atlantic Ceara Rise. *Quat. Sci. Rev.* **25**, 3138-3149, doi:10.1016/j.quascirev.2006.02.011 (2006).

2 Wilkens, R. H. *et al.* Revisiting the Ceara Rise, equatorial Atlantic Ocean: isotope stratigraphy of ODP Leg 154 from 0 to 5 Ma. *Clim. Past* **13**, 779-793, doi:10.5194/cp-13-779-2017 (2017).

3 Zeeden, C. *et al.* Revised Miocene splice, astronomical tuning and calcareous plankton biochronology of ODP Site 926 between 5 and 14.4Ma. *Palaeogeog. Palaeoclimatol. Palaeoecol.* **369**, 430-451, doi:10.1016/j.palaeo.2012.11.009 (2013).

4 Drury, A. J., Westerhold, T., Hodell, D. & Röhl, U. Reinforcing the North Atlantic backbone: revision and extension of the composite splice at ODP Site 982. *Clim. Past* **14**, 321-338, doi:10.5194/cp-14-321-2018 (2018).

5 Billups, K., Pälike, H., Channell, J. E. T., Zachos, J. C. & Shackleton, N. J. Astronomic calibration of the late Oligocene through early Miocene geomagnetic polarity time scale. *Earth Planet. Sci. Lett.* **224**, 33-44, doi:10.1016/j.epsl.2004.05.004 (2004).

6 Pälike, H. *et al.* The Heartbeat of the Oligocene Climate System. *Science* **314**, 1894-1898, doi:10.1126/science.1133822 (2006).

7 Bell, D. B., Jung, S. J. A., Kroon, D., Lourens, L. J. & Hodell, D. A. Local and regional trends in Plio-Pleistocene delta O-18 records from benthic foraminifera. *Geochemistry Geophysics Geosystems* **15**, 3304-3321, doi:10.1002/2014GC005297 (2014).

8 Liebrand, D. *et al.* Cyclostratigraphy and eccentricity tuning of the early Oligocene through early Miocene (30.1–17.1 Ma): Cibicides mundulus stable oxygen and carbon isotope records from Walvis Ridge Site 1264. *Earth Planet. Sci. Lett.* **450**, 392-405, doi:https://doi.org/10.1016/j.epsl.2016.06.007 (2016).

9 Beddow, H. M. *et al.* Astronomical tunings of the Oligocene–Miocene transition from Pacific Ocean Site U1334 and implications for the carbon cycle. *Clim. Past* **14**, 255-270, doi:10.5194/cp-14-255-2018 (2018).

10 Drury, A. J. *et al.* Late Miocene climate and time scale reconciliation: Accurate orbital calibration from a deep-sea perspective. *Earth Planet. Sci. Lett.* **475**, 254-266, doi:10.1016/j.epsl.2017.07.038 (2017).

11 Holbourn, A., Kuhnt, W., Kochhann, K. G. D., Andersen, N. & Meier, K. J. S. Global perturbation of the carbon cycle at the onset of the Miocene Climatic Optimum. *Geology* **43**, 123-126, doi:10.1130/g36317.1 (2015).

12 Tian, J. *et al.* Paleoceanography of the east equatorial Pacific over the past 16 Myr and Pacific–Atlantic comparison: High resolution benthic foraminiferal δ18O and δ13C records at IODP Site U1337. *Earth Planet. Sci. Lett.* **499**, 185-196, doi:10.1016/j.epsl.2018.07.025 (2018).

13 Holbourn, A. *et al.* Middle Miocene climate cooling linked to intensification of eastern equatorial Pacific upwelling. *Geology* **42**, 19-22, doi:10.1130/g34890.1 (2014).

14 Drury, A. J. *et al.* Deciphering the State of the Late Miocene to Early Pliocene Equatorial Pacific. *Paleoceanography and Paleoclimatology* **33**, 246-263, doi:10.1002/2017pa003245 (2018).

15 Holbourn, A. E. *et al.* Late Miocene climate cooling and intensification of southeast Asian winter monsoon. *Nature Communications* **9**, 1584, doi:10.1038/s41467-018-03950-1 (2018).

16 Laskar, J. *et al.* A long-term numerical solution for the insolation quantities of the Earth. *Astronomy & Astrophysics* **428**, 261-285 (2004).

17 Andersson, C. & Jansen, E. A Miocene (8–12 Ma) intermediate water benthic stable isotope record from the northeastern Atlantic, ODP Site 982. *Paleoceanography* **18**, doi:10.1029/2001PA000657 (2003).

18 Cramer, B. S., Toggweiler, J. R., Wright, J. D., Katz, M. E. & Miller, K. G. Ocean overturning since the Late Cretaceous: Inferences from a new benthic foraminiferal isotope compilation. *Paleoceanography* **24**, doi:10.1029/2008PA001683 (2009).

19 Cheng, X., Tian, J. & Wang, P. in *Proceedings of the Ocean Drilling Program, Scientific Results* Vol. 184 (eds W.L. Prell *et al.*) (2004).

20 Amante, C. & Eakins, B. W. *ETOPO1 arc-minute global relief model: procedures, data sources and analysis. NOAA Technical Memorandum NESDIS NGDC-24.* , <doi:10.7289/V5C8276M> (2009).

21 Laskar, J., Fienga, A., Gastineau, M. & Manche, H. La2010: a new orbital solution for the long-term motion of the Earth⋆. *A&A* **532**, A89 (2011).

22 Laskar, J., Gastineau, M., Delisle, J.-B., Farrés, A. & Fienga, A. Strong chaos induced by close encounters with Ceres and Vesta. *Astronomy & Astrophysics* **532**, L4 (2011).
23 Ciais, P. *et al.* in *Climate change 2013: the physical science basis. Contribution of Working Group I to the Fifth Assessment Report of the Intergovernmental Panel on Climate Change* (eds T.F. Stocker *et al.*) 465-570 (Cambridge University Press, 2013).
